# Dr. John O’Reilly’s Prizes

**Published:** 1860-04

**Authors:** John O’Reilly

**Affiliations:** 230 Fourth Street, Washington Square, South


*To the Medical Students of the United States of America.*

I will give a premium of $250 for the Essay which shall be judged the best, by competent judges, on the Anatomy and Physiology of the Animal and Organic Nervous Systems.

The Essays to be sent to me on or before the first of March, 1861.

I will likewise give a second premium of $250 for the best Essay on the same subject.

The Essays to be handed in on or before the first of March, 1862.

The medical students who shall be declared the successful competitors will be required to declare, on their *word* and *honor*, that the Essays are their own productions, and that they have not been assisted by any legally qualified medical man. JOHN O'REILLY, M.D.

230 FOURTH STREET, WASHINGTON SQUARE, SOUTH,
*March 8th*, 1860.

